# T2 weighted three dimensional imaging of the whole heart

**DOI:** 10.1186/1532-429X-11-S1-P216

**Published:** 2009-01-28

**Authors:** Yiu-Cho Chung, Orlando P Simonetti, Renate Jerecic

**Affiliations:** 1 Siemens Healthcare USA, Columbus, OH USA; 2 Ohio State University, Columbus, OH USA; 3 The Ohio State University, Columbus, OH USA; 4 Siemens Healthcare USA, Chicago, IL USA; 5 Siemens Medical Solutions USA, Inc., Chicago, IL USA

**Keywords:** Myocarditis, Slow Heart Rate, Acute Myocarditis, Fast Heart Rate, Heart Imaging

## Introduction

T2 weighted (T2w) dark blood turbo spin echo (DB-TSE) [1] is useful in myocardial tissue characterization such as acute myocarditis and acute myocardial infarction [2, 3]. 3D acquisitions can provide high resolution images of the whole heart, allowing arbitrary views of the organ, and reducing partial volume effect. Conventional 3D TSE improves SNR but not sampling efficiency or spatial resolution. We propose a novel TSE technique for T2w 3D imaging of the whole heart that provides higher spatial resolution and reduces scan time per slice compared to 2D DB-TSE.

## Purpose

To study the feasibility of a T2w 3D TSE technique for whole heart imaging with improved spatial resolution and imaging efficiency compared to 2D DB-TSE.

## Methods

### Sequence

The sequence (aka T2w-SPACE) uses non-selective refocusing pulses [4] to reduce echo spacing and cardiac motion sensitivity. It supports constant (CFL) and variable flip angle (VFL) refocusing pulses. VFL uses longer echo train (ETL) than CFL while maintaining good T2 weighting [4]. Optional motion sensitizing gradient (MSG) was implemented to suppress blood [5]. Navigator gating was supported. The technique was evaluated on a 1.5 T scanner (MAGNETOM Avanto, Siemens, Germany).

### Imaging

T2w-SPACE was first optimized in 5 healthy volunteers (STIR contrast on two of them) using these parameters: inplane voxel = 1.6–1.8 mm isotropic, partition = 2.5–4 mm, 28–40 partitions, 2 averages, TR = 2 heartbeats, bandwidth/pixel = 840 Hz, 100% partition resolution. T2w-SPACE with MSG of 25 mT/m*ms (3 directions) was then compared with 2D DB-TSE in 4 other healthy volunteers using the optimal parameters. SAX views were used. In the comparison, common parameters for 2D and 3D acquisitions were: inplane resolution = 1.8 mm isotropic, fatsat, 100% phase resolution, parallel imaging rate 2 (24 reference lines), and navigator gating. 12 slices and 36 partitions (each 3.2 mm) were acquired by 2D DB-TSE and T2w-SPACE respectively. 2D specific parameters were: slice = 8 mm, ETL = 23, bandwidth/pixel = 300 Hz, echo spacing = 7 ms, TE = 56/76 ms.

## Results

From the 5 volunteers, VFL was preferred in subjects with slow heart rate (RR ≥ 800 ms) while CFL (150°) was suitable for subjects with fast heart rate. Scan times ranged from 2.6 min (VFL, 5.6 s/partitions, 65% navigator efficiency) to 8.2 min (CFL, 13.7 s/partitions, 47% navigator efficiency). T2w-SPACE gave consistently good image quality using parameters in Table 1.

**Table 1:** Optimal imaging parameters for cardiac imaging with T2w-SPACE

Flip angle series	Echo spacing	Echo train (ETL)	Slice turbo factor	Echo train duration	TE
VFL	2.5 ms	85	1	207 ms	115 ms
CFL	2.9 ms	27	2	150 ms	74 ms

**Figure 1:**
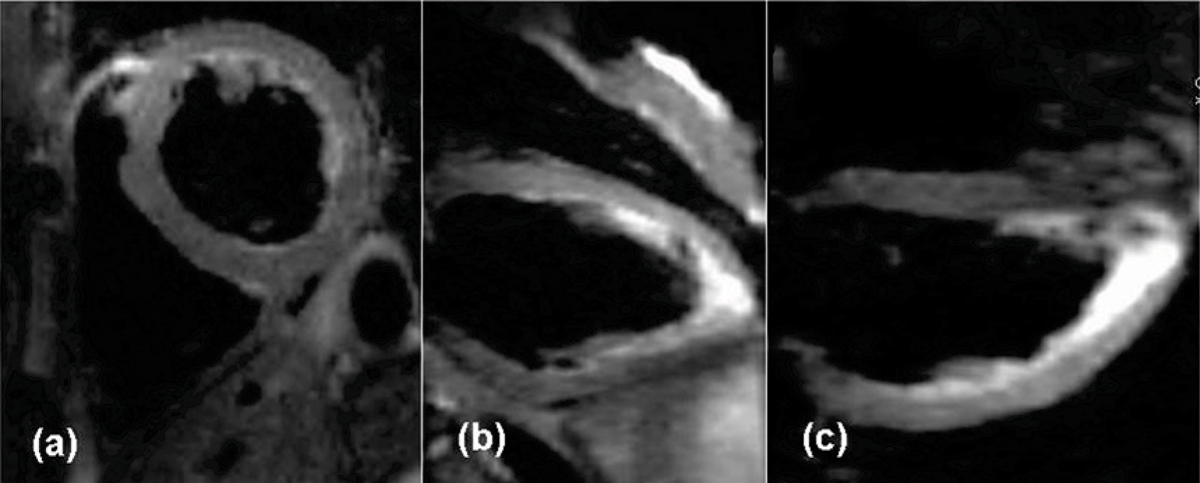
**Three cardiac views of a T2w-SPACE acquisition**.

**Figure 2:**
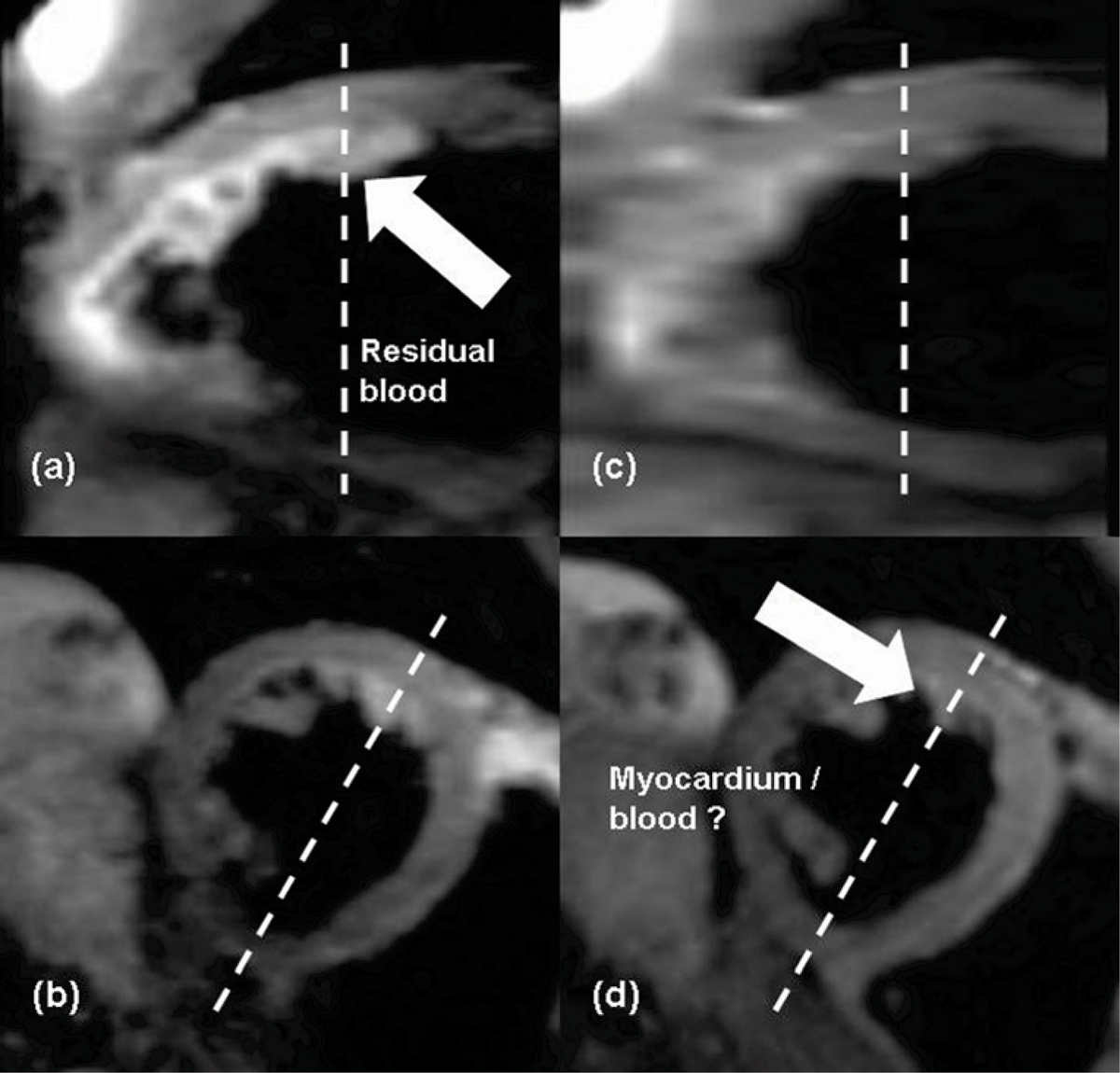
**Partially suppressed blood may mimic myocardium in 2D DB-TSE**. (a) VLA and (b) SAX views from T2w-SPACE, (c) VLA and (d) SAX views from 2D DB-TSE.

In the comparison study, all volunteers had RR ≥ 800 ms, and VFL was used. The echo train in 2D DB-TSE was limited to 161 ms, avoiding excessive T2 decay and signal drop-off. VLA and HLA views can be obtained from T2w-SPACE easily using MPR (Figure 1). Image quality of 2D and 3D images were very comparable except that blood was better suppressed in 2D images. Figure 2 shows comparative images from T2w-SPACE and 2D DB-TSE (co-registered) from one volunteer. Note that the partially suppressed blood may mimic myocardial signal in 2D DB-TSE. Scan time per partition varied from 7.3 sec to 13 sec in T2w-SPACE and from 6 sec to 30 sec in 2D DB-TSE.

## Discussion

T2w-SPACE for whole heart imaging is feasible. It requires 2 and 3 excitations/partition for the VFL and CFL mode respectively and is two times more efficient than 2D DB-TSE, sparing time for improved slice/partition resolution. In reality, heart rate and navigator efficiency determine scan times in both cases. The technique would also be relevant to myocardial tissue characterization at 3 T and beyond.
